# Saponin-Rich Plant Premixture Supplementation Is as Efficient as Ionophore Monensin Supplementation Under Experimental *Eimeria spp* Challenge in Broiler Chicken

**DOI:** 10.3389/fvets.2022.946576

**Published:** 2022-07-14

**Authors:** Mohammed el Amine Benarbia, Pierre Gaignon, Claire Manoli, Pierre Chicoteau

**Affiliations:** ^1^Feed In Tech Join lab, 42 rue Georges Morel, Beaucouzé, France; ^2^Nor Feed, 3 rue Amedeo Avogadro, Beaucouzé, France; ^3^URSE, Ecole Supérieure d'Agricultures, University Bretagne Loire, Angers, France

**Keywords:** cocccidia, Eimeria spp, gut, broiler–chicken, saponin, fenugeek, Yucca (Yucca schidigera)

## Abstract

For decades avian coccidiosis prevention was based on the use of synthetic coccidiostats. However, their intensive use led to the development of resistance phenomena. In addition, societal demand is increasing for antibiotic-free animal products. Thus, there is a need for a natural and efficient solution for coccidiosis management. Saponin-rich plants, like *Yucca schidigera* and *Trigonella foenum-graecum*, are promising tools for coccidiosis management. This study assessed the effects of supplementing broiler chickens with a commercial blend of these two plants (NorponinXO2) under an experimental *Eimeria* challenge and compared their effects to monensin supplementation. Three trials were performed. For each trial, chickens were divided into 4 groups, untreated uninfested control (UUC), infested untreated control (IUC), infested supplemented with 120 ppm of Monensin in feed (PM), and infested supplemented with 250 ppm of Norponin XO2 in the feed (PX). Chickens were raised in cages; experimental infestation was performed on d14. On d21, intestinal lesions (ILs) scores and growth performances were recorded. A statistical study was carried out on each trial, as well as data from the 3 trials. Experimental infestation reduced in a significant way final body weight in IUC broilers compared to UUC broilers. This loss was numerically compensated by PM and PX treatment. As expected, intestinal lesions were almost absent in the UUC group; however, broilers from the IUC group showed a higher intestinal lesion occurrence. Supplementations with Monensin and NPXO were able to reduce intestinal lesions occurrence. These results suggest that NPXO supplementation is as efficient as Monensin in managing coccidiosis.

## Introduction

As broiler production intensified, several breaks in productivity appeared; Coccidiosis is among these breaks. This disease is caused by an apicomplexan parasite of the genus *Eimeria* (1). Animal infestation by the parasite seriously impairs broilers' growth performances (reduced body weight and feed efficiency) and negatively impacts their health status and welfare (2). In addition to negatively impacting the health and welfare of chickens, coccidiosis is a disease with a serious economic impact on poultry producers. This negative impact is linked to the cost of chemoprevention and loss due to decreased animal growth performances (3, 4). In a recent study, Blake et al. estimate the cost of coccidiosis at 0,21US$/bird (5).

The use of synthetic ionophores, like monensin in the chemoprevention of coccidiosis, helped the poultry industry to reach high levels of productivity while preventing coccidiosis (Chapman, 2009). However, this intensive use of these synthetic molecules for decades led to the development of resistant strains of *Eimeria* worldwide (6–10). Moreover, residues of these molecules in animal products and/or the environment are a serious issue (11–13). Facing these new challenges, poultry producers are looking for an efficient tool to add to their global strategy in managing coccidiosis (14). Plant and plant-extract feed additives are among the interesting approaches used to control coccidiosis in broiler flocks (15). The Saponins, thanks to their ability to disrupt cellular membranes, are a promising approach to managing coccidiosis in broiler chickens (16, 17). However, the perception of the effectiveness of these solutions by poultry producers is not always as positive as conventional solutions based on synthetic molecules according to a recent market survey (internal data). The reasons evoked by the professionals during this survey to explain this perception are mainly the rarity of data evidencing their efficacy using usual experimental methods and their mode of action. This highlights the need to generate data using experimental infestation methods to evaluate new solutions for coccidiosis management. The objective of this study was to evaluate and compare the effectiveness of feed supplementation with saponin-rich plant premixture (Norponin XO2®) to ionophore monensin under various experimental *Eimeria* challenges.

## Materials and Methods

### Experimental Design, Products, and Animal Management

Three trials were carried out in three experimental facilities. The same experimental design was applied in each facility. The trials respectively took place in Belgium (Wolvenhof, Poulpharm animal site), the U.S.A (Willington, Colorado, COLORADO QUALITY RESEARCH, INC.), and Spain (Murcia, IMASDE Campus de Espinardo). These three centers were chosen to consolidate the results by maximizing the diversification of the breeding system (chick origin/strain, feed material, and operators). The trials were conducted according to the principles of GCP (2000) Guidelines on Good Clinical Practice for Clinical Trials for Registration of Veterinary Medicinal Products (VICH) and met appropriate current quality standards indicated by European Food Safety Authority. The experimental protocols used in this study were approved by the competent authorities of the country for each trial. Chickens were randomly distributed to either one of four treatments: Untreated Uninfested Control, Infested Untreated Control, Infested and supplemented with recommended inclusion rate in the feed of monensin (120 ppm), Infested and supplemented with the recommended inclusion rate in the feed of Norponin XO2® (250 ppm), and a premixture of saponin-rich plants. Broilers were raised to the age of 21 days in cages. For the supplemented groups, the supplementation started on the first day of the experiment. Feed and water were distributed *ad libitum* for all trials within the 3 experimental facilities. The experimental conditions (sex, strain, the number of animals, the number of replicates, and the size of the cage) are specified in Table 1.

**Table 1:** Specificity of experimental design for each trial.

**Country**	**Spain**	**USA**	**Belgium**
Breed	Ross 308	Cobb 500	Ross 308
Sex	Male	Male & Female	Male
Number of Chicken	160	224	192
Chicken per cage	5	8	8
Light program	18H/24H	24H/24H	18h/24H
Temperature	32°C	32°	32°C

Monensin was purchased from Elanco (Greenfield, Indiana, USA) and Norponin XO2® from Nor-Feed SAS (Beaucouzé, France). The basal diets were formulated to meet or exceed the nutrient requirements recommended by the breed suppliers (Aviagen and Cobb) (Table 2).

**Table 2:** Diet composition and nutrient levels (as-feed basis) according to age and trial.

**Country**	**Belgium**	**Belgium**	**Spain**	**Spain**	**USA**
Feed	Starter	Grower	Starter	Grower	Starter/grower
Age (d)	1–14	15–21	1–14	15–21	1–21
Nutrient Level	
AME_n_ (kcal/kg)	2 958	3 109	3 152	3 266	3150
Crude protein	216.9	201.9	217.6	192.7	220.0
Lysin (g/kg)	12.7	11.9	13.6	12.0	13.5
Met (g/kg)	5.6	5.2	6.3	5.3	7.1
Met+Cys (g/kg)	9.2	8.7	10.0	8.6	10.1
Ca (g/kg)	9.0	7.2	9.0	9.1	9.6
P (g/kg)	6.4	5.4	6.6	6.3	5.9

### Experimental Eimeria Challenge

For the experimental challenge with wild-type *Eimeria spp*, oocytes from the field were used. As *Eimeria. spp* genetic background/virulence was proven to vary depending on geographical region (18), the number of sporulated oocysts per bird in the inoculum was defined by a preliminary dose titration study using susceptible broilers. This preliminary study aimed to determine the number of oocysts needed to obtain infestations leading to homogeneous lesions between the different trials and similar to those encountered in the field. Once the number of sporulated oocysts was determined, broilers were infested with sporulated oocysts in suspension by oral gavage at D14. The number of sporulated oocysts/birds is shown in Table 3. Chicks within the UUC group were gavaged with the same volume of distilled water.

**Table 3:** Inoculum composition.

	***E. acervulina*/bird**	***E. maxima*/bird**	***E. tenella* / bird**
Belgium	52 000	10 000	17 500
Spain	65 000	6 500	15 000
USA	100 00	50 000	75 000

### Data Collection

Broilers were weighed per cage on days 1 and 21 to estimate BW. Feed intake was measured daily. These data were used to estimate average daily gain (**ADG)**, average daily feed intake (**ADFI)**, Feed Conversion Ratio (**FCR**), and European Production Efficiency Factor [**EPEF**, (19)]. EPEF is defined as follows:


(1) $$\begin{array}{r} EPEF=\frac{viability\;\left(\%\right)\times BW\;\left(kg\right)}{age\;\left(d\right)\times FCR\;\left(kg\;feed\;/\;kggain\right)}\;\times 100 \end{array}$$

Dead chicks were counted and removed daily. Broilers were euthanized at 21 days old to proceed to intestinal lesions scoring according to the scoring system published by Johnson and Reid (20).

### Statistical Analysis

Data were analyzed using R (21) with *emmeans* and *nnet* packages. All quantitative data were analyzed using the following model:


(2) $$\begin{array}{r} Y_{ijk}\;=\;Treatment_{i}\;+\;Trial_{j}\;+\;{Treatment:Trial}_{ij}\;+\;\epsilon_{ijk} \end{array}$$

where Y_ijk_ was a dependent variable of a repetition k, within treatment *i* and trial j. Treatment, trial, and their interaction were fixed factors, due to the low number of levels within each factor (22). All observations were weighted according to the number of broilers they represented. Body weight gain (**BWG**), ADG, ADFI, FCR, and EPEF were analyzed using a linear model. Results are reported as means and standard error of the means (mean ± SEM). Individual survival analysis and scores for the intestinal lesions were analyzed using logistic regression, results being reported as odds ratio (**OR**) to the base level and confidence interval (CI) [OR (lower confidence interval—upper confidence interval)]. Values for OR are given in scientific notation as they may be highly variable. The considered base level was IUC treatment for both cases, with a 0 score of lesions and being alive for survival analysis as standard. This allowed us to ensure that inoculation was successful in comparison to UUC treatment, and to assess the effects of both PX and PM treatments. Belgium was randomly chosen as a basal level for the trial effect. Survival analyses were analyzed using the same model as for quantitative data when the interaction was removed for the analysis of lesion scores to avoid error due to levels of scores not being presented for the given trial and treatment. A *P*-value of < 0.05 was used to indicate statistical significance, and between 0.05 and 0.1 was used to indicate a tendency.

## Results

### Growth Performances

Initial BWs were not significantly different between treatments within a trial (P = 0.75 for trial x treatment interaction). The global analysis of from the 3 trials showed that BWs at the end of trials was higher (*P* < 0.05) in the UUC group than in the IUC group, with PX and PM being intermediate but not significantly different from other treatments (*P* > 0.10) (Table 1). The difference at the trial level was only numerical without statistical significance (**Table 5**). There was no significant difference due to treatments either in global analysis or trial level for ADG (P = 0.16), ADFI (P = 0.57), FCR (P = 0.24). or EPEF (P = 0.57) or showing similar growth performances results between treatments (Tables 4, 5). There was a tendency for ADFI (P = 0.10) for chicks within PX treatment to have a lower feed intake in comparison to chicks in PM treatment (48 ± 1.44 g vs. 52.8 ± 1.46 g), but this did not affect BWG or FCR. The mortality rate was very low, around 4%, whatever the considered effects. Survival analysis using logistic regression showed no differences in death OR due to treatment effect (*P* = 0.17), trial effect (*P* = 0.29), or their interaction (*P* = 0.53). This was due to the low number of deaths within each treatment. Survival rates were always higher than 90%, whatever the treatment or the trial.

**Table 4:** Growth performances global analysis.

	**Body Weight Gain (kg)**	**FCR**	**EPEF**
UUC	0,84 ± 0,04 ^a^	1,39 ± 0,02 ^a^	269 ± 15 ^a^
IUC	0,80 ± 0,03 ^b^	1,42 ± 0,02 ^a^	261 ± 12 ^a^
PM	0,82 ± 0,03 ^ab^	1,39 ± 0,02 ^a^	273 ± 15 ^a^
PX	0,81 ± 0,04 ^ab^	1,41 ± 0,03 ^a^	272 ± 16 ^a^

*Values expressed as mean ± SEM ^statisticalgroup^*.

*Body weight gain, FCR, Feed conversion ratio; EPEF, European poultry efficiency factor; UUC, Untreated uninfested control; IUC, Infested Untreated control; PM, Infested monensin treated; PX, infested Norponin XO treated*.

**Table 5:** Growth performance per trial.

		**Body Weight Gain (kg)**	**FCR**	**EPEF**
Belgium	UUC	1,13 ± 0,04 ^a^	1,33 ± 0,02 ^a^	378 ± 20 ^a^
	IUC	1,03 ± 0,02 ^a^	1,35 ± 0,02 ^a^	348 ± 13 ^a^
	PM	1,08 ± 0,03 ^a^	1,30 ± 0,03 ^a^	376 ± 26 ^a^
	PX	1,10 ± 0,02 ^a^	1,33 ± 0,02 ^a^	395 ± 11 ^a^
Spain	UUC	0,76 ± 0,03 ^a^	1,47 ± 0,02 ^a^	219 ± 6 ^a^
	IUC	0,72 ± 0,01 ^a^	1,53 ± 0,03 ^a^	211 ± 8 ^a^
	PM	0,76 ± 0,01 ^a^	1,53 ± 0,03 ^a^	220 ± 7 ^a^
	PX	0,70 ± 0,03 ^a^	1,56 ± 0,05 ^a^	202 ± 13 ^a^
USA	UUC	0,73 ± 0,02 ^a^	1,35 ± 0,01 ^a^	244 ± 8 ^a^
	IUC	0,72 ± 0,01 ^a^	1,36 ± 0,01 ^a^	249 ± 6 ^a^
	PM	0,72 ± 0,01 ^a^	1,33 ± 0,01 ^a^	254 ± 6 ^a^
	PX	0,74 ± 0,02 ^a^	1,35 ± 0,01 ^a^	255 ± 5 ^a^

*Values expressed as mean ± SEM ^statisticalgroup^*.

*Body weight gain, FCR, Feed conversion ratio; EPEF, European poultry efficiency factor; UUC, Untreated uninfested control; IUC, Infested Untreated control; PM, Infested monensin treated; PX, infested Norponin XO treated*.

### Lesion Scores

**Figure 1:**
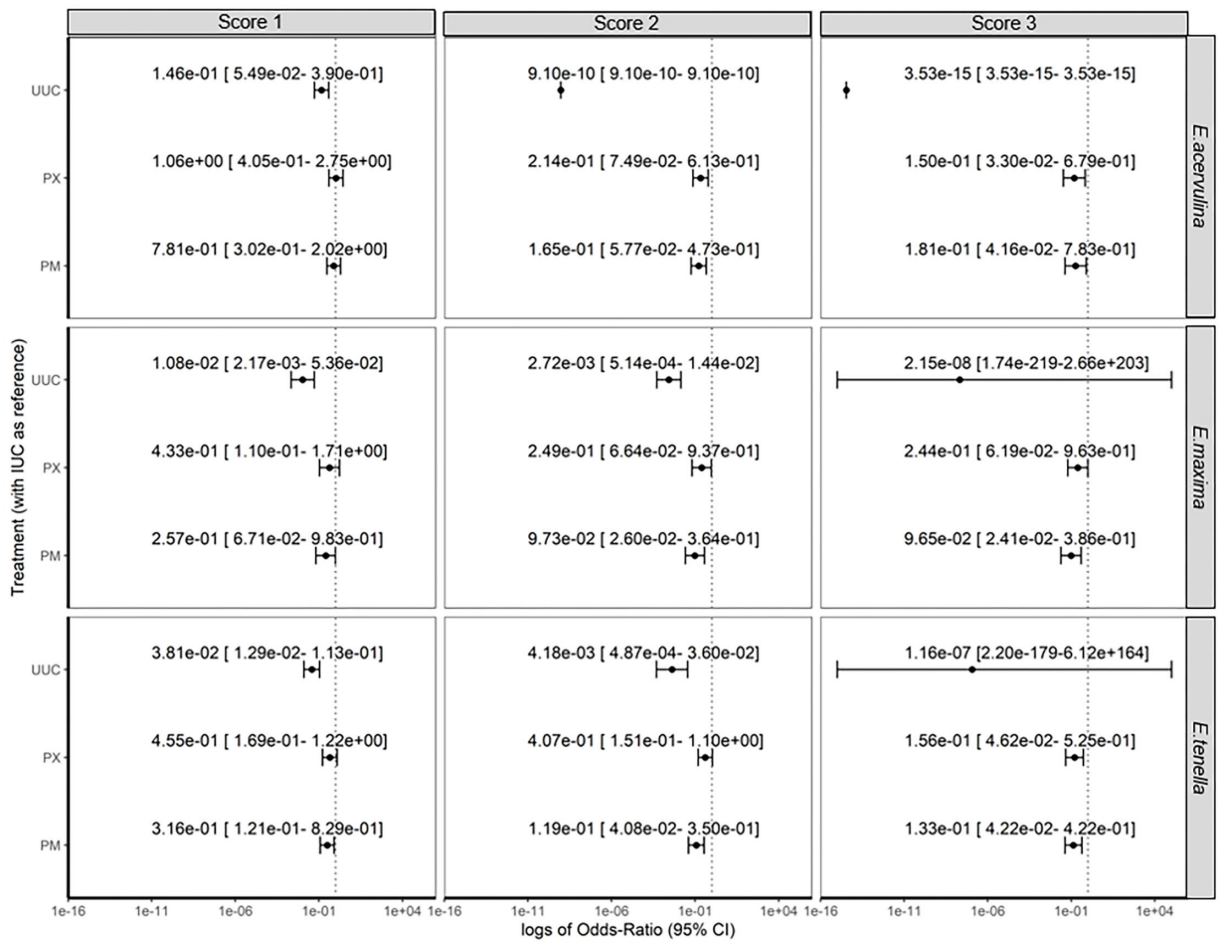
Odds of the apparition of lesions.

Odds of the apparition of lesions (Score 0 vs. Score 1 or higher) were affected by experimental treatments (Figure 1). Apparition of lesions due to *E. acervulina* was significantly lower for the UUC treatment (*P* < 0.001) in comparison to the IUC treatment. The OR was 3.21e-2 [1.25e-2–7.55e-2]. Odds of the apparition of the lesion were similar to IUC treatment for PM and PX groups, with respectively OR of 4.17e-1 [1.77e-1–9.38e-1] and 5.35e-1 [2.25e-1–1.23e+0], even OR for PM treatment was significantly lower. The trial effect was significant (*P* < 0.001), but there was no statistical difference when comparing OR.

Odds of apparition of lesions due to *E. maxima* were lower for UUC, PM, and PX groups in comparison to IUC group, with respective OR of 3.68e-3 [6.91e-4–1.46- 2], 1.30e-1 [3.34e-2–4.22-1] and 2.86e-1 [7.23e-2–9.44e-1]. Trial effects were also highly significant (*P* < 0.01), with higher odds of lesion apparition in the USA and Spain trials, with OR of 8.39e+0 [2.99e+0–2.83 e+1] and 3.89e+1 [1.28e+1–1.53e+2], respectively.

Treatment and trial effects significantly affected the odds of apparition of lesions due to *E. tenella* (*P* < 0.001). Like for the two other types of lesions, UUC treatment had the lowest OR, with a value of 1.52e-2[5.15e-3–3.97 e-2]. PM and PX groups also had lower odds of apparition than IUC group, with respective OR of 1.94e-1 [7.75e-2–4.49e-1] and 3.34e-1 [1.4e-1–8.41e-1]. The USA and Spain trials had higher OR of lesions apparition, with values of 3.01e0 [1.58e0–5.92e0] and 4.49e0 [1.99e0–1.08e1], respectively.

Analysis of the score of the lesions showed significant effects of trial and treatments (*P* < 0.001), whatever the considered type of lesion. Estimations and confidence intervals for each modality of treatment effect and each level of lesions are summarized in Figure 1. UUC treatment always had the lowest OR, whatever the level or type of lesions. For a score of 3+, estimations of UUC treatment were inefficient, infinite, or had a very large confidence interval, as this score was never observed for this treatment, whatever the trial. PM and PX had similar results on the score of lesions, reducing the odds of a higher score, 2 or 3+, in comparison to IUC treatment.

## Discussion

Successful completion of an *Eimeria. spp* challenge is important for the interpretation of treatment results. In this study, we can state that the *Eimeria. spp* challenge was successful as evidenced by the significant decrease in final body weight between infested and non-infested chickens, even though the effects on other performance parameters, FCR, and EPEF, were not statistically significant. Most interestingly, the appearance of intestinal lesions was higher in infested chickens compared to non-infested chickens. The drop in final body weight caused by the experimental challenge in the IUC broilers tended to be compensated by the supplementation of monensin and saponin-rich plant premixture globally. At the trial level, there were some disparities (Table 5). However, this global compensation was only numerical and not statistically significant. It is well-known and documented that feed supplementation with monensin compensates for the loss of performance due to coccidiosis in broiler chicken (23). However, data remain scarce concerning the effect of the saponin-rich plant, *Yucca schidigera*, and *Trigonella foenum greacum* in *Eimeria. Spp* challenge conditions. The few available data report different results and conclusions. Our results are in line with recently published data concerning the use of saponin-rich plants (*Quillaja.s*) by the Bafundo team and those published by Saeed et al. (*Yucca.s*). (24, 25). On the other hand, a recent study shows that saponin-rich plant supplementation (*Yucca.s*) does not have a significant compensation effect on the loss of performance of chickens infested by *Eimeria* (26); thus, contradicting obtained data from the present study. An element that could explain this variability of results could be that the concentration of active compounds in the saponin-rich plants used is different from one study to another. Indeed, the concentration of these active compounds can be more or less high according to several parameters (type of plant, part of the plant used, harvesting period, etc.). In addition, unlike some cited studies, we used a formulation of 2 saponin-rich plants, namely, *Yucca.s* and *Trigonella. f.g.*, thus, making the comparison of the obtained results quite difficult to the available scientific literature. This fact also highlights the importance of transparency and standardization in active compounds when natural plant-based products are used in animal nutrition. (27). The complexity of the parasites and the pathophysiology of coccidiosis infection can also play an important role in the observed variability of the results. In this study, we observed that both treatments (Monensin and Norponin XO2) reduced the appearance of intestinal lesions due to experimental *Eimeria* infestation. If these observations are well-documented for monensin supplementation in chicken (28, 29), data dealing with saponins deserve to be reinforced. The decrease in the occurrence of intestinal lesion scores for the saponin-rich plant treatment can be explained by the direct action of saponins. Indeed, saponins have the property to disrupt the cellular membrane of the parasite, thanks to their permeabilization effects (30). Interacting with the parasite and disrupting its membrane leading to the loss of its homeostasis could be a possible mechanism of action of saponin. Another mechanism of action of saponins can be the inhibition of the invasion step of the parasite. Felici et al. (31) evidenced the fact that saponins can inhibit the invasion process of the parasite. However, the cited studies are mainly *in vitro* studies that did not consider the possible degradation of the natural active compounds of saponin-rich plants. Moreover, in addition to saponin, these plants contain other actives compounds like flavonoids that certainly plays a role in the observed effect on animal and intestinal lesions. Thus, making the investigation of the mechanism of action of saponin-rich plants quite challenging. More studies are needed to better understand the mechanism of action behind the observed effects. Nevertheless, these experimental results showed that a 100% plant-based solution can be as efficient as a conventional coccidiostat in managing coccidiosis; thus, offering more agility for broiler chicken producers. Particulary, studies have shown that the use of alternative solutions to chemoprevention helps to restore the effectiveness of molecules, such as monensin and duclazuril (32, 33). Therefore, introducing a plant-based solution and coccidiosis vaccines could help to solve the resistance problem observed and described all over the world. However, we believe that in addition to the efficient and natural solution, there is a need to rethink coccidiosis management. Parameters like nutritional management (34), biosecurity, and chicken strain selection could help to raise chickens while limiting the use of synthetic and/or ionophore coccidiostats.

This study evidenced the fact that a natural solution formulated from *Yucca schidigera* and *Trigonella foenum-graecum* (Norponin XO2) is as efficient as a monensin supplementation in managing coccidiosis in an experimental-infestation model. These results should be confirmed in the field. Altogether, these elements will certainly help to achieve the ultimate goal of producing a sustainable broiler chicken at a reasonable cost for the continuously growing number of humans on earth.

## Data Availability Statement

The raw data supporting the conclusions of this article will be made available by the authors, without undue reservation.

## Ethics Statement

The animal study was reviewed and approved by IMASDE and POULPHARME Commity.

## Author Contributions

PC and CM reviwed the article. MB wrote the article. Statistical analysis made by PG and CM. Experiments were setup by MB. PC and MB made the experimental design. All authors contributed to the article and approved the submitted version.

## Conflict of Interest

MB and PC work in the R&D departement of Nor Feed commercialize products based on sapoinns. The remaining authors declare that the research was conducted in the absence of any commercial or financial relationships that could be construed as a potential conflict of interest.

## Publisher's Note

All claims expressed in this article are solely those of the authors and do not necessarily represent those of their affiliated organizations, or those of the publisher, the editors and the reviewers. Any product that may be evaluated in this article, or claim that may be made by its manufacturer, is not guaranteed or endorsed by the publisher.
